# *PLOS Genetics* Data Sharing Policy: In Pursuit of Functional Utility

**DOI:** 10.1371/journal.pgen.1005716

**Published:** 2015-12-14

**Authors:** Gregory S. Barsh, Gregory M. Cooper, Gregory P. Copenhaver, Greg Gibson, Mark I. McCarthy, Hua Tang, Scott M. Williams

**Affiliations:** 1 Department of Genetics, Stanford University School of Medicine, Stanford, California, United States of America; 2 HudsonAlpha Institute for Biotechnology, Huntsville, Alabama, United States of America; 3 Department of Biology, The University of North Carolina at Chapel Hill, Chapel Hill, North Carolina, United States of America; 4 Center for Integrative Genomics, School of Biology, Georgia Institute of Technology, Atlanta, Georgia, United States of America; 5 Wellcome Trust Centre for Human Genetics, University of Oxford, Oxford, United Kingdom; 6 Oxford Centre for Diabetes, Endocrinology & Metabolism, University of Oxford, Oxford, United Kingdom; 7 Oxford NIHR Biomedical Research Centre, Churchill Hospital, Oxford, United Kingdom; 8 Department of Genetics, Geisel School of Medicine, Dartmouth College, Hanover, New Hampshire, United States of America

About a year ago, PLOS implemented a new process intended to further the overarching principle that data used in the work we publish should be accessible and reusable. The motivation goes hand-in-hand with both our open access ethos and the scientific method itself: the validity of a conclusion depends on the ability to reproduce the underlying results.

In theory, PLOS’ new data policy, in which “all data underlying the findings described [must be] fully available,” is not so new; in practice, though, it may be perceived as burdensome, complicated, and/or inefficient. The policy and its purpose has been discussed extensively among *PLOS Genetics* Editorial Board members with regard to its potential impact on editors, authors, the community, and research subjects.

The purpose of this editorial is 2-fold: to acknowledge and discuss aspects of the “data-sharing” process that are especially challenging, and to provide additional clarification and guidance in the context of a few scenarios that are especially relevant, all from the perspective of working scientists who read, evaluate, and contribute to research based on genetics and genomics. We also suggest a way forward that builds on what already works at PLOS: consulting with multiple stakeholders whose interests intersect to develop consensus.

An issue that is often problematic for *PLOS Genetics* authors is the sheer volume of data generated by large-scale phenotyping and/or genotyping studies. Whether from a confocal microscope, a radiofrequency detector in an MRI, or a CCD in a DNA sequencing instrument, processing, filtering, and compression of digital data is inherent across many areas of modern biology. It is often neither practical nor wise to archive and distribute the primary output of digital detectors; indeed, the question of what constitutes “raw data” is a moving target.

A second problematic issue arises from ethical concerns associated with human research subjects. The potential to identify research subjects based on genomic information has received considerable attention and has fostered the development of controlled access mechanisms, in which researchers must seek approval from data access committees. As with any new set of regulations, there is a risk of creating more problems than are solved; from an editorial perspective, we recognize that authors must commit significant resources to deposit data into controlled access repositories, and that the process of extracting data can also be problematic. Regulatory burdens also create conflicts since the legitimate concerns of human subject review boards and governmental agencies to protect privacy are not necessarily prima facie aligned with open access policy. In short, the system is not working efficiently.

Finally, for research that is not publicly funded, there can be legitimate reasons to restrict data sharing. Personal genomics companies, agricultural genetics corporations, and domestic animal breeders all support worthwhile research, but may have responsibilities to shareholders and/or contributors that restrict the ability to fully share the underlying data. In addition, there are efforts in some countries to protect data and genetic resources as part of a national interest. While we acknowledge and understand the underlying rationale for governmental and/or commercial restrictions on data sharing, these represent an inherent conflict with the principles of open access.

## Fundamental Principles of Data Sharing at *PLOS Genetics*

Discussions among members of the *PLOS Genetics* Editorial Board, along with consultation with our colleagues at other PLOS journals, have helped us develop guidelines for data sharing dilemmas; in doing so, our goal is to promote functionally useful data sharing while ensuring that the perfect does not become the enemy of the good. The long-term aspiration is to maximize open and comprehensive access to all genotype and phenotype data (and for human subject research, in a manner consistent with ethical guidelines that respect human autonomy). We believe these goals are best accomplished by working together with all participants, including funding agencies, regulatory bodies, and other publishing companies.

First principle: a level playing field. We ask that all authors at *PLOS Genetics* adhere to the same rules when it comes to making data available, regardless of funding source, institutional affiliation, or country of residence. Exceptions will not be made, for example, to allow withholding of data from other academic scientists to further develop a research area or to develop intellectual property.

Second principle: the “rawness” of data appropriate for sharing is determined by how the data has been used, what is needed for meaningful evaluation of the claims being made, and how the data could be used in the future. In other words, “all data underlying the findings described” depends very much on context.

Consider three hypothetical manuscripts: (A) a new technology for massively parallel sequencing or base-calling, (B) a metagenomic survey of a new ecological environment, and (C) a manuscript that describes whole genome sequencing (WGS)-based diagnosis of a rare Mendelian disorder. All three manuscripts entail generation and analysis of billions of DNA sequencing reads, but the nature of the data sharing necessary for the sake of facilitating rigorous evaluation and maximizing impact is very different. For A, in addition to the reads themselves, various “raw-er” data, like images and various stages of intermediate processed data would be needed; for B, all reads, but not the images and pre-processed stages, are likely to be needed; for C, depending on the nature of the analysis and the consent of the samples, shared data might include VCF files relevant to evaluating genotype-phenotype causality.

Third principle: like everything else at *PLOS Genetics*, data sharing is by, and for, working scientists. The goal is to maximize the potential future use of data underlying every manuscript, ensure that we adhere to the highest standards of rigor and criticality, and at the same time, do our best to ensure that data sharing by authors is not overly burdensome. Guidelines for specific scenarios, generated in consideration of the principles articulated above, are provided in a FAQ that will be refined and expanded according to feedback from the community.

## The Data Availability Statement: An Opportunity for Transparency and Evolution of Standards

An important component of PLOS’ process is the Data Availability Statement. At the time of submission, authors are asked to concisely describe what data will be available and how it will be shared; that information is available to editors, reviewers, and, eventually, readers as a component of published manuscripts. We view the statement not as a checkbox to monitor compliance but as an opportunity for authors to state how their plans to share conform to the request for “all data underlying the findings,” how the data will be *functionally useful* to readers and to the community, and to explain any restrictions due to privacy concerns. For the previous hypothetical examples, authors of a sequencing methodology paper (A) might state how they will make available “raw” signals obtained directly from detectors along with routines, examples, and summaries of signal processing needed to convert that signal into base calls; authors of a metagenomic survey (B) might state that all sequence reads that passed quality control metrics for quality and length would be deposited in DRYAD as fastq files. For a WGS-based human disease manuscript (C) in which the consent process allows controlled data sharing of private information, individual VCF files could be made available through the database of Genotypes and Phenotypes (dbGaP) or the European Genome-phenome Archive (EGA), and a focused list of candidate variants relevant to the analysis process (and that would not risk compromising privacy) could be published as a genome coordinate text file in supplementary material.

We are using the data availability statement in two ways. First, at the time of submission, our editorial staff check to see if there are any potential concerns about data availability and, if needed, resolve those concerns before the manuscript enters the formal editorial and review process. The primary goals at this stage are to ensure that sufficient data exists for meaningful review (and therefore avoid potential delays later in the publication process), and to identify situations in which there are restrictions on data sharing that are inconsistent with open access. Second, we now ask reviewers and editors to consider the functional utility of how data is shared when evaluating the potential impact of a manuscript. The underlying rationale is that for many genome-scale studies, data processing, i.e., extraction, curation, formatting, has become a much greater burden than data generation, while at the same time standardized pipelines are increasingly used for many tasks, lessening the need to share "rawer" or intermediately processed data to demonstrate reproducibility. As anyone will testify who has downloaded a supplementary table to begin work on a new analysis only to discover the file is a pdf rather than plain text file, *how* data are shared can be just as important as *what* data are shared. Organizing supporting information in a structured way, or providing data via a structured repository, e.g., GenBank or dbGaP, makes it more likely that the data will be used. Explicit recognition of the importance of functional utility will be facilitated by the data availability statement, and is fully aligned with PLOS’ vision to promote *usability* as well as *availability* of research results.

For example, in a large-scale genotype-phenotype association study, sharing of measurements of Single Nucleotide Variants (SNVs) or transcripts correlated with a trait or perturbed in an experiment would facilitate replication of the specific observations reported in the manuscript. Useful data sharing, however, might go well beyond a focused set of molecular phenotypes to include all measured SNVs and transcripts, so as to facilitate additional or alternative analytical strategies and hypothesis-testing frameworks in the future. For human studies, privacy concerns would likely limit the extent to which all data could be publicly shared, but for studies in other animals or plants, the community will benefit from data sharing strategies that go beyond the ability to simply replicate core findings.

What about experimental organisms? For quantitative comparisons, e.g., tetrad classes, genotype ratios, or fraction of cells exhibiting a particular phenotype, individual-level data that underlies summary statistics depicted in tables or graphs can be made available as supplementary material, providing both an additional level of transparency and the opportunity for others to build on and/or extend an analysis. Regardless of whether authors are working with humans, mice, fish, flies, yeast, or any other organism, the data availability statement should be used to describe explicitly what data is being made available and how.

At the time of submission, editorial staff will use the data availability statement to confirm that the manuscript conforms to our data-sharing policy and that there is sufficient information available to allow meaningful review. Experience to date suggests that ~10% of submitted manuscripts will require communication between the editorial staff and authors to clarify data-sharing issues, and more than half of those situations will be easily resolved. In a minority of situations, communication with a senior editor will be needed, e.g., when potential restrictions on data availability are excessive or not adequately justified, or when there are concerns that data sharing does not meet minimal standards. We expect a concrete data availability statement will facilitate discussion when questions arise at either the “minimal sharing” or “functionally useful” levels and can motivate development and evolution of standards. As a starting point, and with the understanding that it is impossible to articulate a universal set of rules that accounts for every situation, we and other members of the *PLOS Genetics* Editorial Board have developed guidelines for several situations, including those relevant to human genetics, for which questions about privacy and controlled access can be especially thorny. For example, we expect that genome wide association studies will make SNV summary statistics easily and publicly available, and we expect that human disease-focused next-gen sequencing studies will make all specific variants relevant to the conclusions at hand available, via a controlled access mechanism if necessary to protect privacy, and/or public release of aggregate findings, as described further below. Exceptions to these recommendations should be carefully and explicitly justified in the data availability statement, and if they are not, may lead to rejection of a manuscript prior to peer review.

For human genetic studies, the data availability statement should also be used to describe strategies taken to maximize sharing and usability while still protecting privacy. For example, in situations in which the consent prevents public release due to privacy concerns but does allow restricted sharing, deposition into a controlled access repository of individual VCF files and phenotype information is tremendously enabling for future disease gene discovery. In addition, public release of aggregate genotype information (e.g., multiple variants in the same individual are unlinked from one another) as a supplementary file can provide immediate access to readers and users without a formal application and evaluation process, while minimizing the risk of identifiability.

## The Future of Data Sharing

In a science communication utopia, all data would be immediately available without qualification. We see this as the ultimate goal, even if it may be unlikely in the foreseeable future, but there are concrete steps we can take to move in the correct direction.

Three areas merit further discussion. First, as editors, we want to be especially cautious about encouraging, even indirectly, situations in which data is provided only in the context of a legal partnership governing future usage and publication. Part of that caution is exemplified by our principle that all authors be treated equally when it comes to principles and requirements for data sharing. A similar motivation underlies our goal to move away from situations in which data is available from the authors upon request. No matter how well intentioned, such an arrangement can foster inappropriate promises of authorship and disputes over intellectual property, that ultimately, hinder rather than help scientific progress.

For human subject research, as the costs of sequencing continue to fall and the clinical utility of sequencing continues to rise, there may be an inevitable, irreconcilable conflict between privacy and data-sharing. Indeed, as more and more genetic data comes from clinical samples, the prospect of open data sharing of individual level (and hence identifiable) data could become less and less likely. The recent United States National Institutes of Health (NIH) policy on genomic data sharing is a useful touchstone. As of January 25, 2015, NIH "expects that informed consent for future research use and broad sharing *will have been obtained*…" but later clarifies that what is really meant is that subjects *will have been asked*. The eventual impact will depend on how research participants balance the potential advantages of sharing personal data against the risk of compromising one's privacy.

What about controlled access data-sharing for research on human subjects? In theory, data access committees can help safeguard study participants (by restricting access to “qualified researchers”), ensure that research results are used according to terms of the original consent, and protect the needs of community resource data producers to receive recognition (the “Fort Lauderdale agreement”). But there is also a range of requirements, formats, and efficiencies across different repositories and data access committees, and a pressing need to develop new approaches that are effective, responsible, and more unified. Thus, rather than mandate that, i.e., all human subject data used in *PLOS Genetics* publications be deposited in a controlled access repository, we prefer to encourage the genetics community (researchers, publishers, funding agencies, and regulatory bodies) to:

1. Develop strategies that promote release of data that is truly open access.
2. Adopt consent options that allow functionally useful sharing with minimal risks to privacy.
3. Work with other stakeholders to improve controlled access for data that must be restricted.

Looking forward, it has become clear that problems with sharing research results are best served by a shared solution, in which multiple publishers work with the scientists who generate and use the data and the funding agencies who support the work. As the nature and volume of data generation continues to evolve, we must adapt.

